# Depression is associated with delirium after cardiac surgery—a population-based cohort study

**DOI:** 10.1093/icvts/ivac151

**Published:** 2022-05-30

**Authors:** Anna Falk, Jessica Kåhlin, Carolin Nymark, Rebecka Hultgren, Malin Stenman

**Affiliations:** Department of Molecular Medicine and Surgery, Karolinska Institutet, Stockholm, Sweden; Perioperative Medicine and Intensive Care Function, Karolinska University Hospital, Stockholm, Sweden; Perioperative Medicine and Intensive Care Function, Karolinska University Hospital, Stockholm, Sweden; Department of Physiology and Pharmacology, Karolinska Institutet, Stockholm, Sweden; Department of Neurobiology, Care Sciences and Society, Karolinska Institutet, Stockholm, Sweden; Department of Vascular Surgery, Karolinska University Hospital, Stockholm, Sweden; Department of Molecular Medicine and Surgery, Karolinska Institutet, Stockholm, Sweden; Department of Vascular Surgery, Karolinska University Hospital, Stockholm, Sweden; Department of Molecular Medicine and Surgery, Karolinska Institutet, Stockholm, Sweden; Perioperative Medicine and Intensive Care Function, Karolinska University Hospital, Stockholm, Sweden

**Keywords:** Cardiac surgery, Depression, Screening, Patient Health Questionnaire-9, Postoperative delirium, Risk factors

## Abstract

**OBJECTIVES:**

Depression is common in patients with cardiac disease, and preoperative depression is associated with worse outcomes after cardiac surgery. Depression is also correlated with postoperative delirium (POD) after major surgery. However, the association between preoperative depression and POD after cardiac surgery is sparsely studied. The aim of this study was to investigate depression as a predictor for POD in cardiac surgery patients.

**METHODS:**

This population-based cohort study included 1133 cardiac surgery patients in Stockholm 2013–2016. Depression was defined by the Patient Health Questionnaire-9, and POD was evaluated by assessing medical records for symptoms of POD according to Diagnostic and Statistical Manual of Mental Disorders criteria. The association between depression and POD was determined through multivariable logistic regression analysis.

**RESULTS:**

A total of 162 (14%) individuals reported depressive symptoms preoperatively. The incidence of POD was 26% and highest among elderly patients. Among patients with depression, 34% developed POD. In the group of non-depressed patients, 24% developed POD. The overall adjusted odds of delirium were 2.19 times higher in individuals with depressive symptoms compared to controls (95% confidence interval 1.43–3.34). The onset of delirium was most common on Days 0–2 after surgery.

**CONCLUSIONS:**

This unique population-based study in patients undergoing cardiac surgery shows that preoperative depression is associated with POD in a large proportion of treated patients. The findings support the need for improved preoperative screening for depression, especially in younger patients, and enhanced clinical surveillance in the early postoperative period for all patients.

**Figure:**
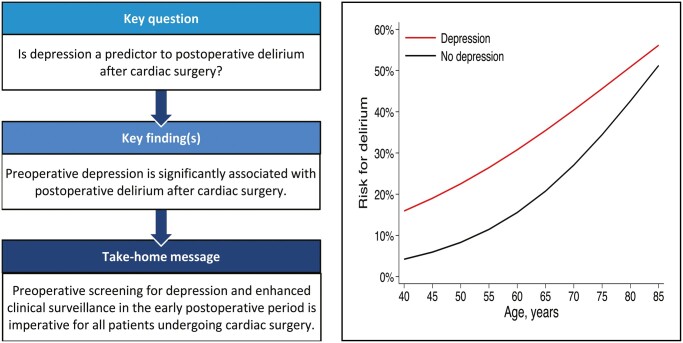

## INTRODUCTION

Depression is a prevalent mental disorder worldwide, in Sweden, the prevalence for clinically significant depression has been reported to be 10.8% [1]. Depression has been associated with increased cardiovascular morbidity and mortality in patients with cardiac disease [2]. Depression screening in patients with cardiovascular disease has therefore been recommended by the American Heart Association [3].

Postoperative delirium (POD) is a serious complication of surgery and patients with POD have poorer long-term outcomes, increased length of hospital stays and often experience reduced quality of life after surgery. The most characteristic symptom of POD is a disturbance in attention that develops over a short period with a tendency to change during the day [4].

Previous studies have demonstrated that depressive symptoms increase the risk for POD in patients 50 years and older undergoing major non-cardiac surgery [5]. Furthermore, depression doubles the risk for POD in patients undergoing spine surgery [6], and this association has also been demonstrated in orthopaedic surgery [7]. However, the correlation between preoperative depression and POD is not yet established for cardiac surgery. In a meta-analysis, an association between preoperative depression and POD was found, but the included studies were of high heterogeneity [8].

The aim of this study was to explore the association between depression and POD in patients undergoing cardiac surgery.

## MATERIALS AND METHODS

### Ethical statement

This study was approved by the human research ethics committee in Stockholm on 30 January 2013, Dnr: 2013/35–31/4. Informed consent was obtained before inclusion.

### Study design

This was a population-based cohort study, based on data from cardiac surgery patients undergoing elective and urgent cardiac surgery. Data were collected at the cardiac surgery department at Karolinska University Hospital between mid-September 2013 and beginning of August 2016 and the inclusion procedure has been described previously [9]. During this period, a depression-screening project was introduced using the Patient Health Questionnaire (PHQ)-9. The Swedish version of PHQ-9 was sent by mail to patients scheduled for elective cardiac surgery ∼2 weeks before their scheduled surgery. Urgent cardiac surgery patients were asked about study participation preoperatively at the regular cardiac surgery ward. Urgent patients were defined as patients scheduled for surgery within 24 h after admission. The patients subsequently received the PHQ-9 prior to surgery from the nurse in charge of patient care. The results from the questionnaires were entered into a study database and further linked to additional baseline characteristics obtained from the Swedish Heart Surgery Register using the unique personal identity number assigned to all Swedish citizens [10]. The study was performed following the STROBE checklist for cohort studies [11].

### Inclusion/exclusion criteria

Swedish speaking patients >18 years old with a planned elective or urgent cardiac surgery at Karolinska University Hospital in Sweden were eligible for inclusion. Patients unable to fill out the PHQ-9 questionnaire prior to surgery were excluded.

### Definitions

Depression is, according to the Diagnostic and Statistical Manual of Mental Disorders (DSM–5), defined when suffering from 5 or more of following symptoms depressed mood with reduced interest in activities, affected appetite, difficulties concentrating, loss of energy, feelings of worthlessness and guilt, thoughts of suicide or suicide attempts [4]. A PHQ-9 score of ≥10 is indicative for clinical depression [12].

Delirium is, according to the DSM–5, defined as inattention, disorganized thinking, and an altered level of consciousness with acute onset and fluctuating course [4].

POD is defined as an onset of delirious symptoms shortly after surgery [13].

Moderate consumption of alcohol is defined as <9 units/week for women and <14 units/week for men and high consumption of alcohol is defined as >9 units/week for women and >14 units/week for men.

Urgent surgery is defined as patients undergoing surgery within 24 h after admission.

### Diagnostic tools and data collection

#### Patient Health Questionnaire

The PHQ is a self-administered, diagnostic, psychometric evaluation tool for depressive symptoms [12]. The PHQ-9 questionnaire consists of 9 questions with answers on a 4-point Likert scale from 0 (not at all) to 3 (nearly every day). A PHQ-9 score ≥10 has a sensitivity of 88% and a specificity of 88% for actual depression [12]. Accordingly, a PHQ-9 score ≥10 was considered indicative of depressive symptoms in this study.

#### Confusion Assessment Method

The proper way to diagnose delirium is through assessment by a psychiatrist using the DSM–5 criteria [4, 14]. However, assessment by a psychiatrist is not feasible in most hospital settings, why numerous delirium assessment tools have been developed to enable bedside diagnosis. The Confusion Assessment Method (CAM) [15] is a standardized instrument, based on the DSM–5 criteria, helping health-care professionals to detect delirium in clinical settings. The CAM-intensive care unit (ICU) is a development of the CAM, modified for use in mechanically ventilated patients in the ICU [15, 16]. The CAM-ICU has high sensitivity, high specificity and high inter-rater reliability and has been translated and psychometrically evaluated for use in Swedish ICU settings [17].

#### Medical records assessment

In addition to using the CAM-ICU, POD could be identified through assessment of clinical symptoms documented in medical records by nurses, physiotherapists, anaesthesiologists and cardiac surgeons. Therefore, medical records were examined by a researcher with extensive clinical experience of intensive care. POD was determined through positive CAM-ICU, International Statistical Classification of Diseases and Related Health Problems code, or by documented symptoms of POD on postoperative Day 0 (day of surgery) to postoperative Day 7 at the ICU and postoperative cardiac surgery ward. To validate the determination of POD, an experienced ICU nurse, working at the department where this study took place, verified 20% of the medical records with a concordance of 90%. In cases of ambiguity, an ICU consultant assisted in determining POD, and any disparity rendered an absence of diagnosis. The presence or absence of POD was compared to the patient’s initial PHQ-9 screening result.

### Surgical management

Cardiac surgery [thoracic aortic surgery, isolated valve surgery, isolated coronary artery bypass grafting and combined procedures] via median sternotomy with cardiopulmonary bypass (CPB) and use of cardioplegia and aortic cross-clamp for full arrest of the heart and a bloodless field, was standard procedure at the department where this study was conducted during 2013–2016.

### Anaesthesiologic procedure and analgesics

Anaesthesia induction with Fentanyl, Propofol and Atracurium was followed by maintenance with Sevoflurane or Propofol, off or on CPB, respectively. Postoperative analgesia with Paracetamol and Morphine or Ketobemidone by patient-controlled analgesia was used as the standard procedure.

### Age groups

To investigate the effect of depression on POD in different age groups, the participants were divided into 3 groups, <62, 62–72 and >72 years. In Sweden, it is possible to start receiving a statutory pension at the age of 62, even though working until the age of 68 is conceivable. The group of patients of <62 years is still of working age. In the group of patients 62–72, there is a mix of employed persons and persons retired due to age. The group of patients of >72 is most likely retired from work.

### Statistical analysis

Baseline characteristics were described with frequencies and percentages for categorical variables, and median values for continuous variables. Student’s *t*-test was used for continuous variables, and the chi-square test was used for categorical variables. Logistic regression was used to estimate odds ratios and 95% confidence intervals (CIs) for the association between depression and POD using non-depression as the reference category. The multivariable model included the following covariates: age, sex, living alone, body mass index, smoking, alcohol use, atrial fibrillation, chronic obstructive pulmonary disease, peripheral vascular disease, percutaneous coronary intervention, prior cardiac surgery, left ventricular ejection fraction, diabetes mellitus, estimated glomerular filtration rate, stroke, EuroSCORE II, type of surgery, CPB, aortic cross-clamp, deep hypothermic circulatory arrest and urgent surgery. Hazard ratios with 95% CI were calculated as relative differences between age groups (<62, 62–72 and >72 years). The statistical analyses were performed with Stata version 16.1 software (StataCorp LP, College Station, TX).

## RESULTS

### Baseline characteristics

**Figure 1:**
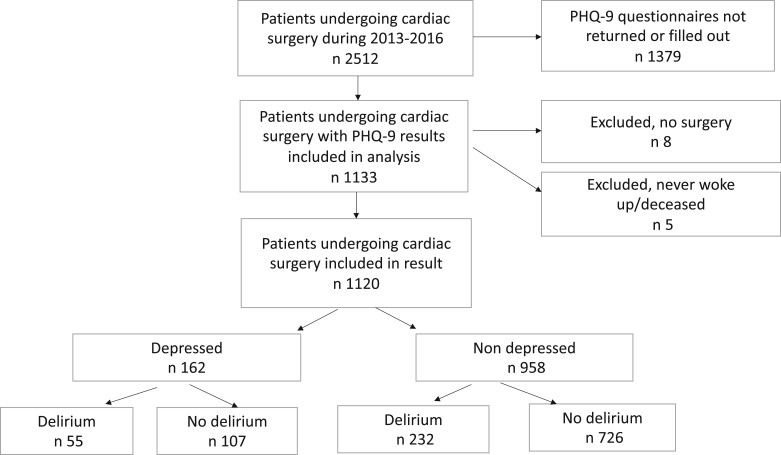
Patient inclusion flowchart. PHQ-9: Patient Health Questionnaire-9.

In the final sample, a total of 1120 patients were included (Fig. 1), 824 (74%) men and 296 (26%) women (Table 1). In the total study population, there were 954 (86%) non-depressed patients, and 162 (14%) depressed patients. The patients were divided into 3 age groups: <62 years (*n* = 373), 62–72 years (*n* = 341) and >72 years (*n* = 406). The mean age at surgery was 65 years old; Among depressed patients, the mean age was 62 vs 66 years old among the non-depressed. Furthermore, depressed patients had chronic obstructive pulmonary disease, were smokers, had higher body mass indexes and consumed alcohol to a greater extent than the non-depressed patients (Table 1).

**Table 1:** Baseline characteristics stratified by depression

	Overall	No depression	Depression	p
No.	1120	958	162	
Age at surgery [mean (SD)]	65 (11.6)	66 (11.4)	62 (12.5)	<0.001
Female sex (%)	296 (26.4)	230 (24.0)	66 (40.7)	<0.001
Living alone (%)	296 (27.7)	254 (27.6)	42 (27.8)	1.000
BMI (%)				0.028
<18.5	10 (0.9)	7 (0.7)	3 (1.9)	
18.5–24.9	395 (35.3)	347 (36.2)	48 (29.6)	
25–29.9	487 (43.5)	421 (43.9)	66 (40.7)	
>30	228 (20.4)	183 (19.1)	45 (27.8)	
Smoking (%)				<0.001
Non-smoker	514 (45.9)	456 (47.6)	58 (35.8)	
Prior smoker	347 (31.0)	290 (30.3)	57 (35.2)	
Current smoker	110 (9.8)	78 (8.1)	32 (19.8)	
Unknown	149 (13.3)	134 (14.0)	15 (9.3)	
Preoperative AF (%)				0.288
No	812 (72.5)	687 (71.7)	125 (77.2)	
Yes	183 (16.3)	163 (17.0)	20 (12.3)	
Unknown	125 (11.2)	108 (11.3)	17 (10.5)	
Preoperative COPD (%)	105 (9.4)	77 (8.0)	28 (17.3)	<0.001
Preoperative PVD (%)	76 (6.8)	62 (6.5)	14 (8.6)	0.397
Preoperative PCI (%)	80 (7.1)	66 (6.9)	14 (8.6)	0.525
Prior cardiac surgery (%)	57 (5.1)	46 (4.8)	11 (6.8)	0.383
LVEF (%)				0.051
>50	752 (67.1)	656 (68.5)	96 (59.3)	
30–50	317 (28.3)	262 (27.3)	55 (34.0)	
<30	51 (4.6)	40 (4.2)	11 (6.8)	
Preoperative DM (%)	184 (16.4)	149 (15.6)	35 (21.6)	0.071
eGFR (%)				0.594
>60	923 (82.4)	785 (81.9)	138 (85.2)	
45–60	136 (12.1)	119 (12.4)	17 (10.5)	
<45	61 (5.4)	54 (5.6)	7 (4.3)	
Preoperative stroke (%)	81 (7.2)	65 (6.8)	16 (9.9)	0.215
Alcohol (%)				<0.001
Never	100 (8.9)	68 (7.1)	32 (19.8)	
Moderate consumption	931 (83.1)	826 (86.2)	105 (64.8)	
High consumption	42 (3.8)	28 (2.9)	14 (8.6)	
Unknown	47 (4.2)	36 (3.8)	11 (6.8)	
Type of surgery (%)				0.016
Isolated CABG	300 (26.8)	251 (26.2)	49 (30.2)	
Isolated valve	515 (46.0)	431 (45.0)	84 (51.9)	
Other	305 (27.2)	276 (28.8)	29 (17.9)	
CPB [mean (SD)]	119.5 (61.0)	118.7 (59.1)	124.6 (71.9)	0.308
XCL [mean (SD)]	84.9 (44.7)	83.99 (42.9)	90.9 (54.4)	0.105
DHCA (%)	64 (5.7)	59 (6.2)	5 (3.1)	0.169
Urgent surgery (%)	139 (12.4)	116 (12.1)	23 (14.2)	0.537
EuroSCORE II [mean (SD)]	3.00 (3.95)	2.96 (3.88)	3.26 (4.32)	0.376

AF: atrial fibrillation; BMI: body mass index; CABG: coronary artery bypass grafting; COPD: chronic obstructive pulmonary disease; CPB: cardiopulmonary bypass; DHCA: deep hypothermic circulatory arrest; DM: diabetes mellitus; eGFR: estimated glomerular filtration rate; LVEF: left ventricular ejection fraction; PCI: percutaneous coronary intervention; PVD: peripheral vascular disease; XCL: aortic cross-clamp.

### Prevalence and onset of postoperative delirium

**Figure 2:**
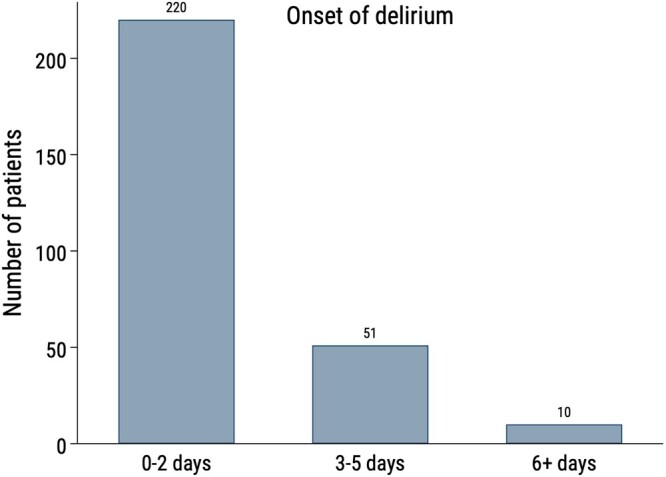
Onset of postoperative delirium. Information about onset of postoperative delirium was missing for 6 patients.

In the total study population, POD was detected in 287 of 1120 patients (26%) and the majority, 220 of the 287 (77%), developed POD during the first 2 days after surgery, as demonstrated in Fig. 2. The incidence of POD was similar between men and women (214/824, 26% of men and 73/296, 25% of women). Transmission from ICU to the cardiac surgery ward was most common on the first day after surgery when 77.5% (*n* = 868) of the patients were transferred from ICU.

### Depression and postoperative delirium related to sex and age

**Figure 3:**
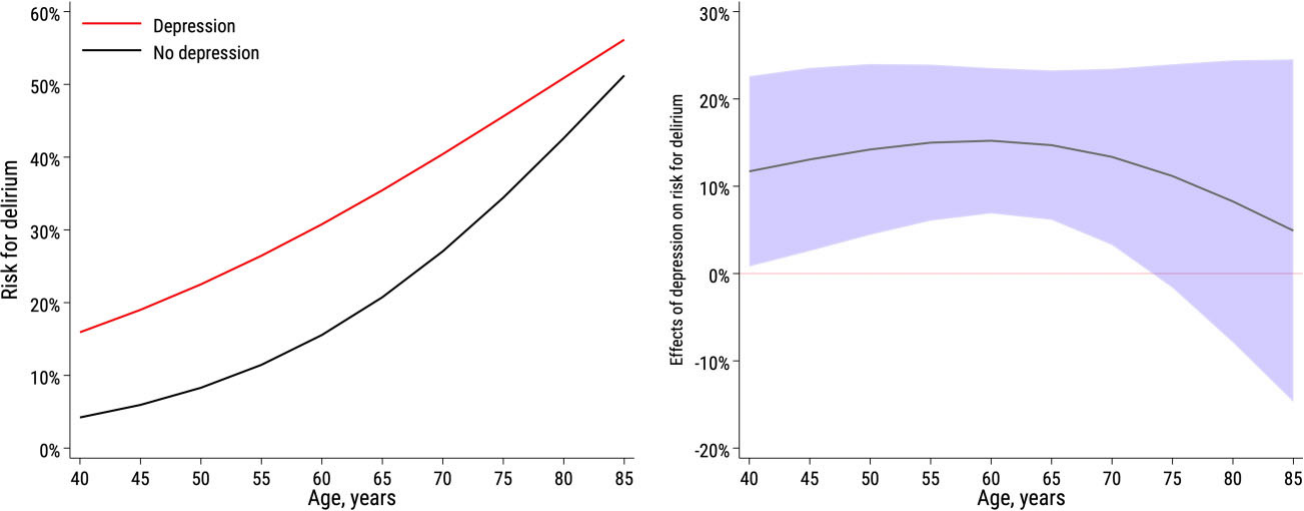
Effect of depression on risk for postoperative delirium.

The risk difference for POD between depressed and non-depressed patients is shown in Table 2 and Fig. 3. Depressed women had a higher risk of developing POD compared to non-depressed women (Table 2). Separate analyses in subsets of patients according to age showed that the risk for POD was highest among the oldest patients (>72 years), where 151/406 (37%) developed POD. However, depressed patients below 62 years had a significantly higher risk of developing POD compared to non-depressed patients below 62 years (Table 2).

**Table 2:** Crude risk of delirium in patients with or without depression according to sex and age

		No. (%) with delirium			Risk difference %	Risk ratio
	No. of patients	Depr−	Depr+	*P*-value	(95% CI)	(95% CI)
Total population	1120	232 (24%)	55 (34%)	0.009	9.7% (2.0 to 17.5%)	1.40 (1.10 to 1.79)
Sex						
Men	824	182 (25%)	32 (33%)	0.080	8.3% (−1.6 to 18.3%)	1.33 (0.98 to 1.82)
Women	296	50 (22%)	23 (35%)	0.029	13.1% (0.4 to 25.8%)	1.60 (1.06 to 2.42)
Age group						
<62 years	373	32 (11%)	19 (26%)	0.001	15.4% (4.7 to 26.0%)	2.44 (1.47 to 4.05)
62–72 years	341	70 (23%)	15 (35%)	0.106	11.4% (−3.6 to 26.4%)	1.49 (0.94 to 2.35)
>72 years	406	130 (36%)	21 (46%)	0.207	9.5% (−5.7 to 24.8%)	1.26 (0.90 to 1.78)

CI: confidence interval.

### Association between depression and postoperative delirium

Among patients with depression, 34% developed POD. In the group of non-depressed patients, 24% developed POD. Depressed patients under 62 years of age have a 15.4% increased risk of developing POD compared to non-depressed patients (Table 2). A depressed 60-year-old patient has the same risk of developing POD after cardiac surgery as a non-depressed 75-year-old patient (Fig. 3). The effect of depression on POD decreases with age (Fig. 3).

In the total study population, there was a crude association between depression and POD: odds ratio (OR) 1.61 (95% CI 1.13–2.30). When divided into age categories, the crude association between depression and POD was strongest among the youngest patients (<62 years): OR 2.95; (95% CI 1.56–5.58; Table 3). In the multivariable model, preoperative depression was associated with POD (OR 2.19; 95% CI 1.43–3.34), compared with no preoperative depression. Most apparent was the strong association between depression and POD in patients below 62 years of age (OR 3.76; 95% CI 1.70–8.32; Table 3).

**Table 3:** Association between depression and delirium in the total study population and by age groups

	Odds ratio (95% CI)		
	Crude	Adjusted for sex and age	Multivariable adjusted
Total population	1.61 (1.13–2.30)	2.28 (1.54–3.36)	2.19 (1.43–3.34)
Sex			
Men	1.50 (0.95–2.37)	2.21 (1.35–3.63)	2.11 (1.23–3.61)
Women	1.93 (1.06–3.49)	2.38 (1.27–4.47)	2.43 (1.13–5.21)
Age group			
<62 years	2.95 (1.56–5.58)	3.03 (1.60–5.84)	3.76 (1.70–8.32)
62–72 years	1.74 (0.88–3.45)	1.87 (0.93–3.75)	2.06 (0.91–4.63)
>72 years	1.49 (0.80–2.76)	1.56 (0.83–2.92)	1.67 (0.80–3.49)

Multivariable analysis adjusted for baseline characteristics: age, sex, living alone, BMI, smoking, alcohol use, AF, COPD, PVD, PCI, prior cardiac surgery, LVEF, DM, eGFR, stroke, EuroSCORE II, type of surgery, CPB time, XCL time, DHCA and urgent surgery.

AF: atrial fibrillation; BMI: body mass index; CI: confidence interval; COPD: chronic obstructive pulmonary disease; CPB: cardiopulmonary bypass; DHCA: deep hypothermic circulatory arrest; DM: diabetes mellitus; eGFR: estimated glomerular filtration rate; LVEF: left ventricular ejection fraction; PCI: percutaneous coronary intervention; PVD: peripheral vascular disease; XCL: aortic cross-clamp.

## DISCUSSION

One out of 4 patients undergoing cardiac surgery developed POD in the analysed sample, and a strong association between preoperative depression and POD was detected in this population-based cohort study, suggesting that preoperative depression is a predictor of POD in patients undergoing cardiac surgery in our region. Moreover, we found that patients younger than 62 years of age with depression prior to surgery suffered almost 4 times the risk of developing POD compared to non-depressed patients in the same age group. These new and clinically relevant insights, on the specifically increased risk for younger patients and patients with preoperative depression, should presumably contribute to alterations in the care trajectory of cardiac patients in general when undergoing cardiac surgery.

Prior studies in various surgical fields have associated preoperative depression with an increased risk of POD. Elsamadicy *et al.* [6] presented depression as an independent risk factor for POD after elective spine surgery and Koskderelioglu *et al.* [7] found that depression was strongly associated with POD in patients >65 undergoing hip surgery. In line with these studies on general surgery, our study on cardiac surgery patients demonstrated that adult patients with symptoms of depression prior to surgery were more likely to develop POD after cardiac surgery compared to patients without a history of depression. The depressed patient group has different perioperative prerequisites, with cardiac disease, combined with cardiac surgery, as well as supporting CPB, which entails a separate risk for embolic cerebral events [18]. This study reveals that the effect of depression on the risk for POD is of great importance in patients <62 years old undergoing cardiac surgery, but that this effect decreases with age. A possible explanation as to why the association between depression and POD was strongest among the youngest patients in this study could be that elderly patients undergoing cardiac surgery have several other comorbidities and therefore are more vulnerable to anaesthesia, surgery and CPB, making the impact of depression less significant. Another explanation for the association between depression and POD is the profound inflammatory response to surgery and CPB that has been considered to contribute to POD in patients undergoing cardiac surgery [19]. It may be argued that the inflammatory response caused by CPB [20] and major surgery could be more critical to patients with an already activated inflammatory response due to depression [21]. In our study, the onset of POD occurred primarily during the first 2 days after surgery, which is in line with results from Zhang *et al.* [22] where POD was diagnosed during the first 2 days after coronary artery bypass grafting [22]. However, this timing of POD is earlier than described by Sugimura *et al.* [23] and Kazmierski *et al.* [24], who reported onset of POD on postoperative Days 3–4 after cardiac surgery. A possible explanation for our results could be the routine of extubating patients early, which might increase the possibility of early detection of POD. About 30–40% of POD cases are considered preventable [25]. In perioperative care, it is essential to identify patients at risk of developing POD, rapidly diagnose POD, and effectively manage patients with POD [13]. Immediate treatment of cause factors and symptoms has a large impact on reducing the duration of POD and thereby improving the prognosis [13, 19]. We found that patients <62 years old with depression were at an increased risk for POD. These patients could benefit from the same multi-component POD preventive intervention as that recommended for the elderly, especially during the first 2 days after surgery, as this period is particularly critical. POD is associated with negative consequences, such as cognitive decline, reduced quality of life, hospital readmissions, increased costs and increased mortality [26]. Therefore, prevention and early detection of POD should be prioritized in all cardiac surgery patients, particularly the elderly and patients suffering from depression.

### Strengths and limitations

The prospectively collected information about depression, the large patient cohort and the procedure by which the evaluation of POD was performed, all add to the strength of this study. Limitations that should be addressed include the response rate of the PHQ-9 questionnaire. The total response rate was 45% (64% among elective patients and 15% among urgent patients). This could have underestimated our results, as other studies report a depression frequency of 20–45% in patients with heart disease. Furthermore, data about POD were collected retrospectively and there was a lack of consecutive delirium assessment instruments at the cardiac surgery ward at the time of the study, it is therefore possible that all symptoms of POD were not documented. The assessments made were performed by nurses, physiotherapists, anaesthesiologists and cardiac surgeons involved in routine daily care instead of a psychiatrist using a delirium assessment tool. Furthermore, data regarding depressive symptoms were self-reported. Since only 15 patients in the total study population were treated with anti-depressants, we decided to exclude information about anti-depressant medication use in this study. Another potential bias is that scheduled patients filled out the PHQ-9 2 weeks before surgery in contrary to the urgent surgery patients who filled out PHQ-9 within 24 h before surgery. We adjusted for urgent surgery in the multivariable analysis.

### Implications

Our study reveals a need for preoperative depression screening for all cardiac surgery patients, as recommended by the American Heart Association. Furthermore, structured assessment tools for POD, such as CAM, recommended for cardiac surgery patients, should be implemented in the early postoperative period for all cardiac surgery patients.

## CONCLUSIONS

This unique population-based study in patients undergoing cardiac surgery shows that preoperative depression is associated with POD in a large proportion of treated patients. The findings support the need for improved preoperative screening for depression, especially in younger patients, and enhanced clinical surveillance in the early postoperative period for all patients.

## Funding

This work was supported by the Mats Kleberg foundation 2021-049 (M.S.), Swedish Society of Medicine Project Grant (J.K.) and European Society of Anesthesia and Intensive Care Research Support Grant (J.K.).

**Conflict of interest:** none declared.

### Data availability

The data underlying this article cannot be shared publicly due to the privacy of individuals that participated in the study. The data will be shared on reasonable request to the corresponding author.

### Author contributions

**Anna Sofia Falk:** Conceptualization; Data curation; Formal analysis; Methodology; Writing—original draft; Writing—review & editing. **Jessica Kåhlin:** Data curation; Funding acquisition; Supervision; Writing—review & editing. **Carolin Nymark:** Supervision; Writing—review & editing. **Rebecka Hultgren:** Supervision; Writing—review & editing. **Malin Stenman:** Conceptualization; Data curation; Formal analysis; Funding acquisition; Methodology; Project administration; Supervision; Writing—review & editing.

### Reviewer information

Interactive CardioVascular and Thoracic Surgery thanks Carlos A. Mestres, Milan Milojevic and the other anonymous reviewer(s) for their contribution to the peer review process of this article.

## Notes

These authors take responsibility for all aspects of the reliability and neutrality of the data presented and its discussed interpretation.

## ABBREVIATIONS

CAM: Confusion Assessment Method
CAM-ICU: Confusion Assessment Method for the Intensive Care Unit
CI: Confidence interval
CPB: Cardiopulmonary bypass
DHCA: Deep hypothermic circulatory arrest
DSM–5: Diagnostic and Statistical Manual of Mental Disorders
ICU: Intensive care unit
PHQ: Patient Health Questionnaire
POD: Postoperative delirium
